# Somatic *NLRP3* mosaicism in Muckle-Wells syndrome

**DOI:** 10.1186/1546-0096-13-S1-P61

**Published:** 2015-09-28

**Authors:** E Gonzalez-Roca, A Mensa-Vilaro, S Plaza, MC Anton, J Rius, E Ruiz-Ortiz, JM Campistol, A Souto, J Cañellas, K Nakagawa, R Nishikomori, J Yagüe, JI Arostegui

**Affiliations:** 1 Hospital Clinic, Immunology, Barcelona, Spain; 2 Hospital Clinic, Nephrology, Barcelona, Spain; 3 Hospital Universitario Santiago de Compostela, Rheumatology, Santiago de Compostela, Spain; 4 Hospital Universitari Germans Trias i Pujol, Rheumatology, Badalona, Spain; 5 Kyoto University, Pediatrics, Kyoto, Japan

## Introduction

Familial cold autoinflammatory syndrome, Muckle-Wells syndrome (MWS), and chronic, infantile, neurological, cutaneous and articular (CINCA) syndrome are dominantly inherited autoinflammatory diseases associated to gain-of-function *NLRP3* mutations. All these diseases are currently considered as different phenotypes of the cryopyrin-associated periodic syndromes (CAPS). A variable degree of somatic *NLRP3* mosaicism has been recently detected in ≈35% of patients with CINCA. However, no data are currently available regarding the relevance of this genetic mechanism in other CAPS phenotypes.

## Objective

To evaluate somatic *NLRP3* mosaicism as the disease-causing mechanism in patients with CAPS phenotypes other than CINCA and *NLRP3* mutation-negative by conventional, Sanger-based genetic studies.

## Materials and methods

*NLRP3* analyses were performed by Sanger sequencing and by targeted deep sequencing. Apoptosis-associated Speck-like protein containing a CARD (ASC)-dependent nuclear factor kappa-light chain enhancer of activated B cells (NF-κB) activation and transfection-induced THP-1 cell death assays determined the functional consequences of the detected variants.

## Results

32 Spanish patients fulfilling clinical inclusion criteria were enrolled. A variable degree (9.4-34.9%) of somatic *NLRP3* mosaicism was detected in 9.3% of enrolled patients (3/32). Their clinical phenotypes were identical to that seen in MWS. Three different missense variants (p.D303A, p.L411F and p.F523L) were identified, being two novels (p.D303A and p.L411F). Bioinformatic and functional analyses confirmed that they were disease-causing, gain-of-function *NLRP3* mutations. Treatment with anti-IL-1 drugs showed long-lasting and positive clinical and biochemical responses.

## Conclusion

We herein show novel evidence about the role of somatic *NLRP3* mosaicism in MWS pathogenesis, which probably represents a shared genetic mechanism in CAPS not restricted to CINCA syndrome. The data here described allowed us to achieve the definitive diagnoses of these patients, which have had serious clinical implications such as gaining access to anti-IL-1 treatments under legal indication and genetic counseling. The detection of somatic gene mosaicism is difficult when using conventional methods. Potential candidates should benefit from the use of novel technologies such as targeted deep sequencing.

